# Parathyroid hormone related peptide and receptor expression in paired primary prostate cancer and bone metastases

**DOI:** 10.1038/sj.bjc.6600115

**Published:** 2002-02-01

**Authors:** A A G Bryden, J A Hoyland, A J Freemont, N W Clarke, N J R George

**Affiliations:** Christie Hospital, Wilmslow Road, Manchester M20 4BX, UK; Department of Osteoarticular Pathology, University of Manchester, Stopford Building, Oxford Road, Manchester M13 9PT, UK; Department of Urology, Withington Hospital, Nell Lane, Manchester M20 2LR, UK

**Keywords:** prostate cancer, parathyroid hormone-related peptide, bone metastases

## Abstract

Parathyroid hormone-related peptide is a regulatory protein implicated in the pathogenesis of bone metastases, particularly in breast carcinoma. Parathyroid hormone-related peptide is widely expressed in primary prostate cancers but there are few reports of its expression in prostatic metastases. The aim of this study was to examine the expression of parathyroid hormone-related peptide and its receptor in matched primary and in bone metastatic tissue from patients with untreated adenocarcinoma of the prostate. Eight-millimetre trephine iliac crest bone biopsies containing metastatic prostate cancer were obtained from 14 patients from whom matched primary tumour tissue was also available. Histological grading was performed by an independent pathologist. The cellular location of mRNA for parathyroid hormone-related peptide and parathyroid hormone-related peptide receptor was identified using *in situ* hybridization with ^35^S-labelled probe. Expression of parathyroid hormone-related peptide and its receptor was described as uniform, heterogenous or negative within the tumour cell population. Parathyroid hormone-related peptide expression was positive in 13 out of 14 primary tumours and in all 14 metastases. Receptor expression was evident in all 14 primaries and 12 out of 14 metastases. Co-expression of parathyroid hormone-related peptide and parathyroid hormone-related peptide receptor was common (13 primary tumours, 12 metastases). The co-expression of parathyroid hormone-related peptide and its receptor suggest that autocrine parathyroid hormone-related peptide mediated stimulation may be a mechanism of escape from normal growth regulatory pathways. The high frequency of parathyroid hormone-related peptide expression in metastases is consistent with a role in the pathogenesis of bone metastases.

*British Journal of Cancer* (2002) **86**, 322–325. DOI: 10.1038/sj/bjc/6600115 www.bjcancer.com

© 2002 The Cancer Research Campaign

## 

Despite increasing detection and treatment of early prostate cancer, this disease still accounts for 9000 deaths annually in England and Wales (Office for National Statistics, 1998). The development of bone metastases is undoubtedly a major cause of morbidity and mortality; patients presenting with osseous involvement have a mean survival of just 2.5 years (George, 1988).

The overwhelming majority of prostate cancer bone metastases are osteoblastic, characterized by increased rates of lamellar bone resorption and replacement with abnormal woven bone. Therefore, in common with osteolytic metastases, osteoclast activation is a key step in the pathogenesis of osteoblastic bone metastases (Clarke *et al*, 1991, 1993). Consequently osteo-active cytokines and growth factors produced by cancer cells may influence the tropism of certain tumours for metastasis to bone, and offer an advantage in the genesis of bony secondaries.

Parathyroid hormone related peptide (PTHrP) is a protein with N-terminal homology to PTH (Guise *et al*, 1997). It is able to bind to and activate the PTH receptor and is consequently a potent stimulant of osteoclasts and osteoblasts (Abou-Samra *et al*, 1992). Work in breast carcinoma has suggested that PTHrP may be an important factor in bone metastasis. Serum PTHrP levels are elevated in patients with bone metastases (Bundred *et al*, 1991) and that there is up regulation of PTHrP expression in the metastatic tissue (Powell *et al*, 1991). Additionally there are also found to be high levels of expression of the receptor for PTHrP in breast cancer bone metastases (Downey *et al*, 1996).

PTHrP is expressed in benign prostate, although its function is unclear (Cramer *et al*, 1996). In primary prostate cancer PTHrP staining intensity increases with tumour grade (Asadi *et al*, 1996), and *in vitro* cell line studies have suggested that it may be a significant autocrine growth factor (Iwamura *et al*, 1994a). Drawing on the work in breast cancer and the parallels between the two tumours, it has been suggested that PTHrP may be a potential factor in the pathogenesis of prostate cancer bone metastases (Guise, 1997).

It is hypothesized that expression of PTHrP and PTHrP receptor (PTHrPr) offer an advantage in the genesis of bone metastases in prostate cancer through paracrine and autocrine mechanisms. The aim of this study was to test this hypothesis by investigation of the co-expression of PTHrP and PTHrP receptor in matched pairs of untreated primary prostate cancer and their corresponding bone metastases.

## MATERIALS AND METHODS

Having obtained informed consent, iliac crest bone biopsies were taken from men known or strongly suspected to have untreated metastatic prostate cancer from whom primary prostatic tissue was also available. Local Ethics Committee approval was granted for the study. All specimens were fixed in formalin and the bone biopsies decalcified in EDTA. All tissue was embedded in paraffin wax. On analysis of the bone specimens 14 were found to contain metastatic prostate cancer. All primary and metastatic tumours were graded according to the Gleason system (Gleason and Mellinger, 1974) by an independent pathologist.

The expression of PTHrP and PTHrPr were determined by *in situ* hybridization on 7 μm tissue sections. The cDNA probes were kind gifts to the Department of Osteoarticular Pathology from Dr MT Gillespie (St Vincent's Institute for Medical Research, Victoria, Australia) and Dr E Schipani (Massachusetts General Hospital, MA, USA) respectively.

### Probe labelling

Radioactive labelling of the probe using ^35^S was achieved using the Amersham Megaprime™ DNA labelling system. The probe and primer were mixed, then the double stranded DNA probe denatured by immersion in a boiling bath. The labelling reaction was initiated by mixing the denatured DNA, reaction buffer, unlabelled deoxynucleotide triphosphate, ^35^S-deoxycytosine triphosphate, and Kenlow fragment of DNA polymerase. Following incubation at 37°C for 1 h, the reaction was stopped by the addition of 0.2 M EDTA pH 8.0. The labelled probe was then purified by centrifugation.

### Prehybridization

Following dewaxing, tissue sections mounted on sialane coated slides were subjected to the following procedures. Tissue permeabilization was carried out by incubation with proteinase K for 1 h (5 μg ml^−1^ for prostate and 10 μg ml^−1^ for bone sections). Control sections were created by elimination of the hybridization signal by incubation for 2 h at 37°C with RNase A. Post-fixation was carried out in 0.4% paraformaldehyde at 4°C.

### Hybridization

The hybridization buffer containing the ^35^S-labelled probe was applied to all tissue sections and then incubated overnight at 37°C.

### Post-hybridization washes

A series of progressively higher stringency washes were performed, using 0.5×SSC/1 mM EDTA/10 mM Dithiolthreitol, then 0.5×SSC/1 mM EDTA, and then in 50% formamide : 50% 0.15 M NaCl/5 mM Tris pH 7.4/0.5 mM EDTA pH 8.0, (SSC – standard saline citrate solution: 7.5 mM NaCl/0.75 mM NaCitrate). Final washes were then performed in 0.5×SSC, initially at 55°C then at room temperature. Sections were dehydrated in industrial methylated spirit and air dried.

### Detection

Binding of the radiolabelled probe was detected by autoradiography. After exposure at 4°C for 2–3 weeks to a layer of Ilford K5 emulsion, slides were developed using Ilford D-19 developer, and fixed with Ilford Hypam. Sections were counter stained with Mayer's haematoxylin, dehydrated and mounted with Xam.

### Analysis

Precipitation of the silver granules produced by autoradiography was assessed using a Leitz Laborlux 12 microscope with the ×20 objective lens, with transmitted light and dark field condensers. The distribution of signal across the tumour cell was described as uniform, heterogeneous or negative.

## RESULTS

Only three of the primary tumours were moderately differentiated, the remainder being poorly differentiated (Gleason ⩾8). All metastases were poorly differentiated. The distribution of tumour grades are shown in Table 1

**Table 1:**
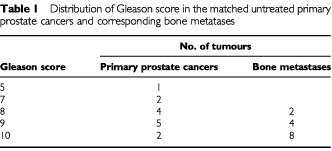
Distribution of Gleason score in the matched untreated primary prostate cancers and corresponding bone metatases

.

**Figure 1:**
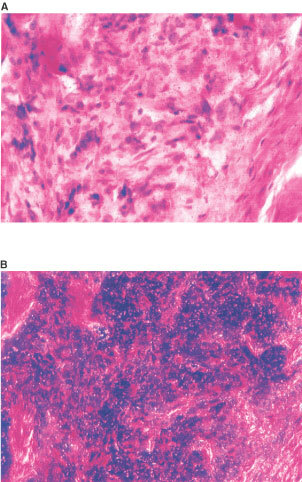
Section of prostatic bone metastasis having undergone *in-situ* hybridization with the probe for PTHrP receptor. Micrograph (**A**) standard light ground field, stained with H&E, and micrograph (**B**) is the same section but with dark ground field, and the silver grains show as the bright spots. The distribution of the bright spots over the metastatic cells indicate uniform signal for PTHrP receptor.

PTHrP expression was positive in 13 out of 14 primary tumours and in all 14 metastases. PTHrP expression was greater in the metastases than the primary tumours. Uniform staining was detected in 11 cases, three metastases exhibited heterogeneous staining, and in no case was PTHrP staining negative. Receptor expression was evident in all 14 primaries and 12 out of 14 metastases. The majority of primary tumours (85%) had uniform expression, while seven and five metastases had uniform (Figure 1

) and heterogeneous staining respectively. In eight cases there was similar expression levels of PTHrP receptor in the paired tumours, while in six cases there was down regulation of receptor expression in the metastatic prostate cells. The patterns of PTHrP and PTHrP receptor expression in the specimens are summarized in Table 2

**Table 2:**
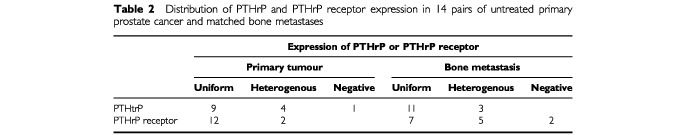
Distribution of PTHrP and PTHrP receptor expression in 14 pairs of untreated primary prostate cancer and matched bone metastases

.

## DISCUSSION

Animal models of bone metastasis have demonstrated the importance of PTHrP in the metastatic cascade. The activation of osteoclasts by PTHrP in conjunction with interleukin 1 has been shown to be essential for bone metastasis development (Arguello *et al*, 1988). In mice inoculated with highly metastatic breast and lung cancer cell lines, treatment with an anti-PTHrP antibody, reduced both tumour burden and osteoclastic bone resorption (Guise *et al*, 1996; Iguchi *et al*, 1996).

PTHrP has been most extensively investigated in breast carcinoma, where 60% of primary tumours are found to have positive expression of PTHrP (Southby *et al*, 1990; Bundred *et al*, 1992). In metastases in soft tissues only 17% are positive for PTHrP in contrast to 92% of those in bone (Powell *et al*, 1991). These findings support the view that PTHrP has a role in the development and progression of bone metastases.

Although PTHrP is expressed in normal prostate (Cramer *et al*, 1996), benign prostatic epithelial cells do not show enhancement of growth in the presence of PTHrP in tissue culture (Peehl *et al*, 1997). PTHrP is secreted by all three commonly studied human malignant prostatic cell lines (PC3, DU-145, and LNCaP) and levels are highest in the bone metastasis derived PC3 line (Iwamura *et al*, 1994a). The same study demonstrated that culture with synthetic PTHrP led to increased proliferation of PC3 and DU-145 cells (Iwamura *et al*, 1994a), suggesting that PTHrP may be a significant autocrine factor in prostate tumour growth, particularly in bone metastases.

PTHrP expression in primary prostatic carcinoma is common (Iwamura *et al*, 1993; Lee *et al*, 1997). Lymph node metastases exhibit similar levels of PTHrP expression to those of poorly differentiated primary tumours (Asadi *et al*, 1996). In this study mRNA for PTHrP was detected in all 14 primary prostate tumours, although expression was heterogeneous in four cases. In the corresponding bone metastases, all 14 were positively staining, all but three being uniformly stained. Previous studies of 10 (Bryden *et al*, 2002) and 14 (Iddon *et al*, 2000) archival prostatic bone metastases by immunohistochemistry have demonstrated PTHrP expression in only 50% of samples. Differences in the technique of PTHrP detection are unlikely to account for this, as concordance between immunohistochemistry and *in-situ* hybridization for detection of PTHrP has been described (Iwamura *et al*, 1994b) suggesting that PTHrP expression is regulated at the gene level. This difference may be due to variation in PTHrP expression with tumour grade as described for primary prostate carcinoma (Iwamura *et al*, 1993). In our previous study 7 out of 10 specimens contained well or moderately differentiated tumour (Bryden *et al*, 2002), and lower levels of PTHrP expression might be anticipated than in the exclusively poorly differentiated group of prospectively collected metastases. In the series investigated by Iddon *et al* (2000), neither the differentiation of the metastases, nor their treatment status (untreated, hormone manipulated or escaped hormonal control) is described, and it is therefore difficult to comment given these variables. It must also be considered that the sample sizes are small and that the differences in levels of PTHrP expression might simply represent true variation.

Despite the proposed importance of PTHrP in the development of prostatic bone metastases, paradoxically, hypercalcaemia is found in less than 2% of patients. In contrast to this, hypercalcaemia is seen in up to 20% of breast cancer sufferers with bone metastases (Coleman and Reubens, 1987). Hypercalcaemia may be partially accounted for by the release of bone bound calcium from lytic deposits, and conversely in the sclerotic metastases of prostate cancer there is calcium ‘bone hunger’ for new bone formation, which may even lead to secondary hyperparathyroidism (Minisola *et al*, 1987). However the humoural contribution of PTHrP to calcium homeostasis derangement is well described in breast cancer, and the detection of PTHrP is strongly correlated with hypercalcaemia (Bundred *et al*, 1991). Few studies have investigated the serum levels of PTHrP in prostate cancer specifically but levels have been found to be similar to those in normocalcaemic patients without malignancy (Kao *et al*, 1990). This would be consistent with the variable secretion of PTHrP into the serum from tumours in which PTHrP can be detected (Dunne *et al*, 1993). Consequently the autocrine/paracrine effects of PTHrP are not universally coupled to the humoural effects.

Staining of the same specimens for the receptor for PTHrP was positive in all 14 primary prostate tumours and expression was heterogeneous in only two cases. In the bone metastases, 12 of 14 stained positively, but in six staining was heterogeneous. The role of the PTHrP receptor has not previously been described clearly in benign or malignant human prostate tissue. Immunohistochemistry has failed to demonstrate the PTHrP receptor in the canine model of benign prostate (Blomme *et al*, 1998), which would be consistent with the lack of growth factor response to PTHrP found in benign prostate by Peehl *et al* (1997).

The high levels of PTHrP receptor demonstrated in this study in primary (100%) and metastatic (85%) prostate cancer are similar to those described in breast carcinoma, where PTHrP receptor can be detected in 61–96% of tumours (Carron *et al*, 1997; Downey *et al*, 1997; Iezzoni *et al*, 1998) using *in-situ* hybridization or PCR. There are no comparable data for prostate cancers available. However for PTHrP to have an autocrine or paracrine effect, it is logical that the PTHrP receptor would need to be expressed within the tissue. PTHrP does not appear to act as a growth factor in benign tissue (Peehl *et al*, 1997), consequently it might be hypothesized that development of PTHrP receptor expression is a significant component of the malignant phenotype.

Overall, this study has shown a high level of expression of PTHrP and its receptor in bone metastases of prostate cancer and their corresponding primary tumours. This supports the previously described work in prostate and other tumour systems, proposing that PTHrP is a likely mediator predisposing to the formation of bone metastases. The co-expression of PTHrP and its receptor would suggest that PTHrP is able to act as an autocrine and/or paracrine growth factor in prostate cancer.

Further work to define and investigate the expression of the PTHrP receptor in benign and malignant prostatic tissue would be important to determine whether this is a feature exclusive to malignant prostatic cells and whether there is any temporal relationship to the presence of bone metastases.
